# Wetting Properties of Clathrate Hydrates in the Presence of Polycyclic Aromatic Compounds: Evidence of Ion-Specific Effects

**DOI:** 10.1021/acs.jpclett.2c01846

**Published:** 2022-08-25

**Authors:** Anh Phan, Michail Stamatakis, Carolyn A. Koh, Alberto Striolo

**Affiliations:** † Department of Chemical and Process Engineering, Faculty of Engineering and Physical Sciences, University of Surrey, Guildford, Surrey GU2 7XH, United Kingdom; ‡ Department of Chemical Engineering, University College London, London WC1E 7JE, United Kingdom; § Center for Hydrate Research, Chemical & Biological Engineering Department, Colorado School of Mines, Golden, Colorado 80401, United States; ∥ School of Chemical, Biological and Materials Engineering, University of Oklahoma, Norman, Oklahoma 73019, United States

## Abstract

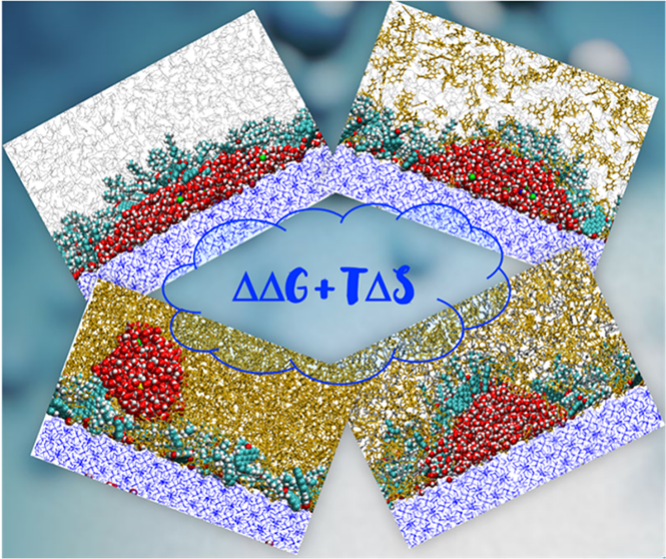

Polycyclic aromatic hydrocarbons (PAHs) have attracted remarkable multidisciplinary attention due to their intriguing π–π stacking configurations, showing enormous opportunity for their use in a variety of advanced applications. To secure progress, detailed knowledge on PAHs’ interfacial properties is required. Employing molecular dynamics, we probe the wetting properties of brine droplets (KCl, NaCl, and CaCl_2_) on sII methane–ethane hydrate surfaces immersed in various oil solvents. Our simulations show synergistic effects due to the presence of PAHs compounded by ion-specific effects. Our analysis reveals phenomenological correlations between the wetting properties and a combination of the binding free-energy difference and entropy changes upon oil solvation for PAHs at oil/brine and oil/hydrate interfaces. The detailed thermodynamic analysis conducted upon the interactions between PAHs and various interfaces identifies molecular-level mechanisms responsible for wettability alterations, which could be applicable for advancing applications in optics, microfluidics, biotechnology, medicine, as well as hydrate management.

Due to their condensed structure and tendency to form π–π stacking configurations,^1,2^ polycyclic aromatic hydrocarbons (PAHs) are emerging as promising candidates for electronic, optical, sensing, energy-storage, and biomedical applications.^2−6^ As opposed to engineered carbon materials, such as carbon nanotubes^7−9^ and graphene,^10−13^ only a few pioneering investigations have so far explored the potential of PAH-based materials, which are abundant and affordable, as they naturally occur in gasoline, crude oil, and coal, for applications in reverse electrodialysis,^14^ electronics, optoelectronics,^15^ and energy storage.^16^ The wide-ranging structural and chemical heterogeneity of PAHs yields complex nanostructure networks with inherent functional and chemical diversity,^17,18^ which could be beneficial for tuning the wettability of PAH-based materials for microfluidics, manufacturing, and heat transfer applications.^3,5,19^ However, it should be noted that PAHs can be of concern for human health and also for the environment.^20,21^

Promoting advances in technologies such as those listed above requires a better understanding of interfacial properties in the presence of PAHs. Hence, in this work we quantify the effect of self-assembled PAH structures on the wettability of clathrate hydrates, systems which have significant direct relevance for preventing the formation and agglomeration of hydrate particles with brine droplets.^22−25^ Although several studies considered model asphaltenes, members of the PAHs family, at liquid–liquid and solid–liquid interfaces,^26−32^ very little is known about the molecular-level interactions between PAHs and hydrate interfaces. To fill this knowledge gap, we implement atomistic molecular dynamics (MD) simulations to examine the wetting behavior of brine droplets (KCl, NaCl, and CaCl_2_) on sII methane–ethane (C1–C2) hydrates coated with PAHs and immersed in oil solvents. The simulation results show significant differences in wetting properties observed for different brine droplets. Inspired by Prausnitz’s commentary^33^ in reference to the well-known book by Lewis and Randall,^34^ we invoke the “*broad highway of thermodynamics*” to explain the new phenomena of hydrate wettability observed here, with the ambition of pioneering a growing field. In particular, we attempt to explain differences in wetting behavior observed as a function of salt and solvent type by conducting advanced thermodynamic analyses, including binding free-energy and configurational entropy changes upon solvation.

In Figure 1A, we present the model PAH molecule considered in this work, Violanthrone-79, which has a single polyaromatic core, aliphatic chains, and functional groups with heteroatoms, such as oxygen. This compound was chosen because it is available commercially, enabling the possible experimental verification of the results presented here. To construct the C1–C2 hydrate surface coated with PAHs, we first prepared the hydrate substrate with *X*, *Y*, and *Z* dimensions of 10.386, 5.193, and 1.731 nm, respectively, following our previously published procedures.^23,35^ More details on the construction of hydrate substrates are reported in the Supporting Information (SI). Then, we placed 28 PAH molecules on top of the hydrate surface, and we equilibrated the system at 274 K and 3.45 MPa until equilibrium. Equilibrium was considered achieved when the system energy as well as density profiles of solvents along the *Z* direction perpendicular to the hydrate surface converge. We present the 2D density profiles obtained for PAHs adsorbed on the hydrate surface in Figure 1B to illustrate their interfacial distribution.

**Figure 1:**
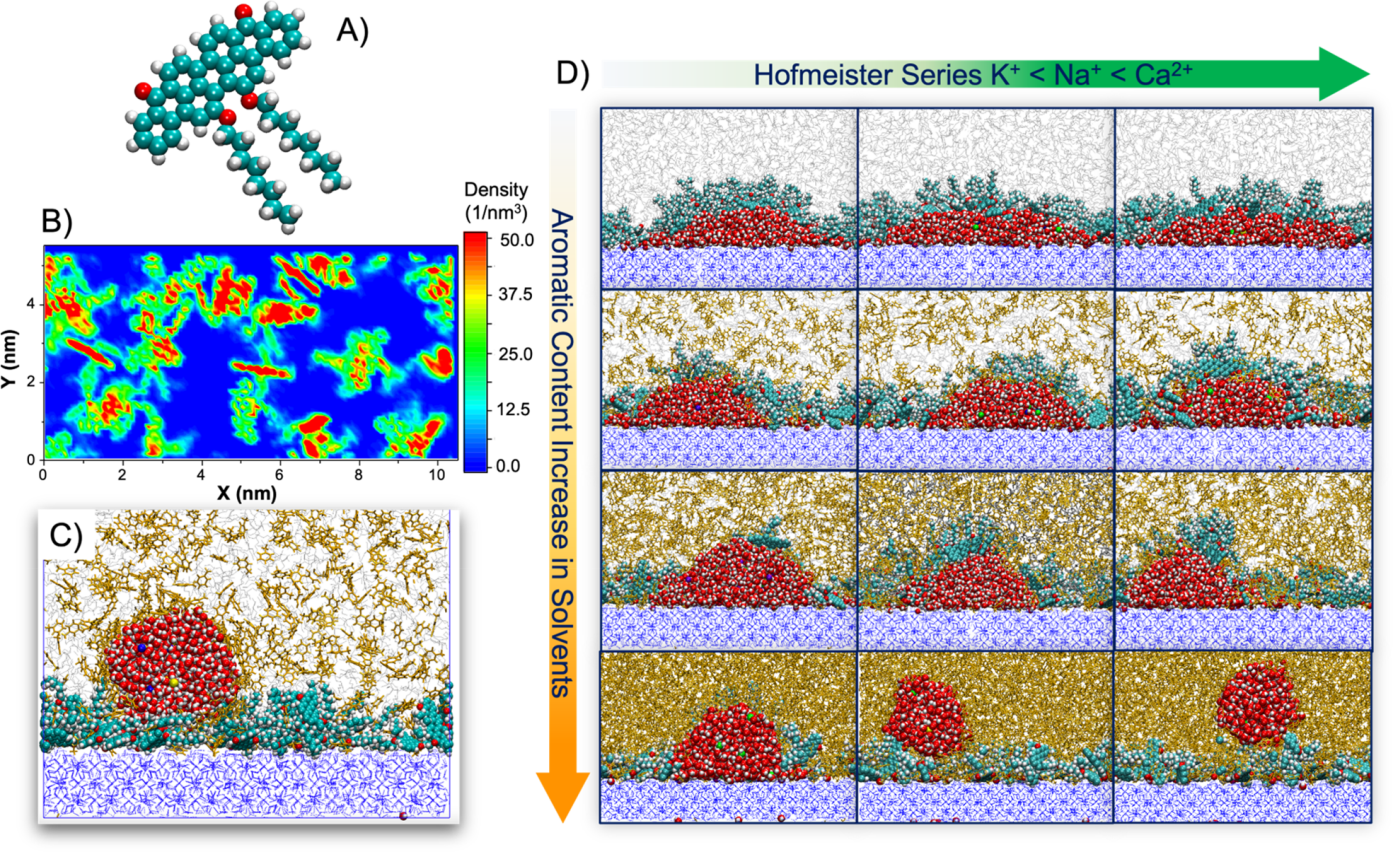
(A) Molecular model of the PAH molecule used in this work, Violanthrone-79. (B) 2D density profile obtained for PAHs adsorbed on the hydrate surface at the beginning of the simulations. (C) Schematic representation of the initial configuration for a brine droplet on a PAH-covered hydrate surface immersed in an organic solvent at 274 K and 3.45 MPa. (D) Simulation snapshots illustrating the final configurations for various brine droplets (3.5 wt %) on PAH-covered hydrate surfaces at varying solvent compositions (from top to bottom, the volume fraction of toluene in heptane–toluene mixtures increases from 0% to 25%, 50%, and 100%). Red, white, and cyan spheres represent oxygen, hydrogen, and carbon atoms, respectively. Green, purple, yellow, and blue spheres symbolize chloride (Cl^–^), potassium (K^+^), sodium (Na^+^), and calcium (Ca^2+^) ions, respectively. Gray and yellow spheres represent heptane and toluene in the oil solvents. Blue wireframes symbolize water in the hydrate while methane and ethane molecules trapped in the solid hydrate structure are not shown for clarity.

Once the substrate was prepared, aqueous ∼3.5 wt % KCl, NaCl, and CaCl_2_ cylindrical droplets (3.5 wt % is the salt concentration of seawater),^36^ periodic along the *Y* direction, were situated on top of the solid substrate. The simulation box was then filled with organic solvents, prepared by varying the heptane–toluene relative composition (see Figure 1C). In our previous studies,^35,37,38^ we confirmed that the system size used here is sufficiently large to minimize system-size effects on the estimated contact angles. Employing atomistic MD simulations, each system was simulated at 274 K and 3.45 MPa for 500–700 ns. Once equilibrium was achieved (see Figure 1D), system properties such as 2D density profiles for water molecules were determined (see the insets in Figure 2) and used to extract the wetting properties of interest (i.e., contact angles). Additional details regarding simulation models, algorithms, methods, and computational procedures are reported in the SI.

**Figure 2:**
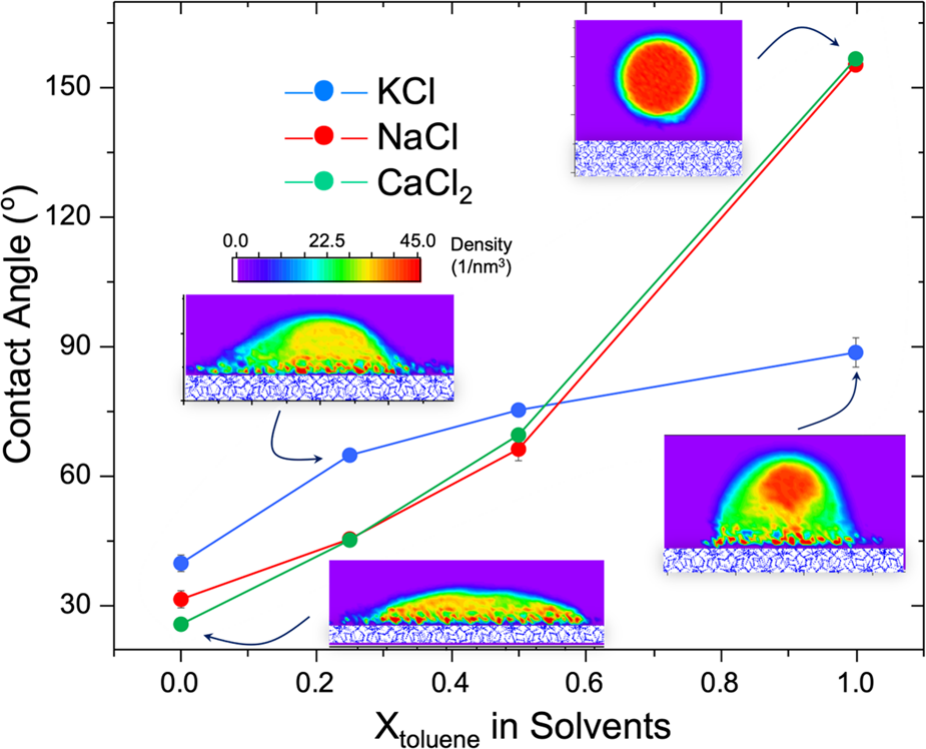
Contact angles estimated for brine droplets on the C1–C2 hydrate surface covered with PAHs. The results were obtained for aqueous ∼3.5 wt % KCl (blue), NaCl (red), and CaCl_2_ (green) droplets in solvents of varying toluene volume fraction (see Table S1). The insets show 2D density profiles of various brine droplets. The color bar shows water density in the units of 1/nm^3^. Error bars, which were obtained from three independent simulation runs for contact angle estimation, are shown, but most are smaller than the symbols used to display the data.

We extracted contact angles from the 2D density profiles obtained for the simulated brine droplets (see insets in Figure 2) following previous procedures.^35,37,38^ Details regarding the methods are reported in the SI. Our simulation results show that the contact angle increases when the toluene content in the solvent is increased (see Figure 2). Strong ion-specific effects are also evident. The contact angle for the KCl droplet is larger than those for NaCl and CaCl_2_ droplets when the toluene volume fraction is below 0.5, although the rate of increase thereof is suppressed upon further increasing toluene content. The contact angles for NaCl and CaCl_2_ droplets are similar for all solvent compositions considered. It is worth noting that these droplets have very large contact angles (>150°) when the solvent is pure toluene, in which case our simulations, in some circumstances, reveal spontaneous rolling of the brine droplets on the PAH-covered hydrate surface (see details in Movie S1 in the Supporting Information). In this latter case, the contact angle was estimated when the droplet resided in one position at the PAH-covered surface (see snapshots in Figure 1D, bottom right). This phenomenon (rolling off) is not observed for the KCl droplet, which yields a contact angle of 90° when it is in direct contact with the hydrate surface. This suggests that synergistic effects due to PAH, aromatic solvents, and salt-specific effects can induce superhydrophobicity on the clathrate hydrate surfaces.

Our results complement literature reports. For example, investigating ion effects on surfaces covered by self-assembled monolayers terminated with benzene, Rimmen et al.^39^ showed that Ca^2+^ and Na^+^ are unlikely to associate with aromatic rings because of the strong hydration, while K^+^ binds strongly to benzene, interacting with up to three aromatic rings at once, in good agreement with other studies.^40−43^ Lisy et al.^42^ discovered a structure of Na^+^(C_6_H_6_)_8_(H_2_O)_4_ with an inner shell of four water molecules and an outer layer of eight benzene molecules, while Williams et al.^43^ reported that six water molecules provide an adequate shielding that prevents benzene from closely interacting with Ca^2+^. These self-assembled clusters in effect lead to an aqueous–aromatic phase separation at the nanoscale.^43^ Similar phenomena could explain why NaCl and CaCl_2_ droplets located on the PAH-covered hydrate maintain their cylindrical shape in pure toluene, while the KCl droplet gradually penetrates the adsorbed PAH–toluene film, reaching the hydrate surface, and ultimately yielding a contact angle of ∼90°.

To put these results in perspective, it helps observing that, when PAHs are not present, our simulations reveal insignificant differences in the contact angles obtained for brine droplets on the bare hydrate surface immersed in solvents, even pure toluene, for which an aqueous–aromatic phase separation would be expected in the presence of Na^+^ and Ca^2+^ ions (contact angle of ∼36°) (more details in the SI, Figure S1). Similarly, we also observe the negligible differences in the contact angles obtained for water droplets on the hydrate surface covered with PAHs in the absence of salt ions (see Figure S2 in the Supporting Information).

One fundamental question arises from the quantitative analysis above: what mechanisms are responsible for the remarkable differences in the wetting behaviors of the brine droplets shown in Figure 2? To address this question, we conduct detailed thermodynamic calculations because an interplay of PAHs, aromatic content in solvents, and salt-specific effects seems to prompt different observations for hydrate wettability.

In Figure 3A, we present potential of mean force (PMF) profiles experienced by one PAH molecule as it approaches the brine–oil interface along the direction perpendicular to the interface from the hydrocarbon phase. The results are shown for various solvents, e.g., pure heptane (blue), heptol50 (50:50 heptane/toluene) (red), and pure toluene (green). We considered only brines containing KCl (empty circles) and NaCl (filled circles) as the wetting behaviors of NaCl and CaCl_2_ droplets on the hydrate surfaces are similar for all solvents simulated (Figure 2). The PMF profiles were obtained as functions of the distance *l* between the center of mass of the PAH molecule and the interfaces. The results, in general, show an effective attraction between PAH and the interfaces. We observe negligible salt-specific effects. On the other hand, the PMF profiles strongly depend on the solvent composition; specifically, the PAH experiences much stronger attraction to the pure heptane/brine interface compared to the other interfaces, whereas the attraction is slightly more pronounced for heptol50 than for toluene. This difference in behavior seems to be comparable with experimental observations^44^ according to which heptane causes asphaltene precipitation, whereas toluene keeps the asphaltenes dissolved in the solvent (note that Violanthrone-79 is often used as model asphaltene^32,45^).

**Figure 3:**
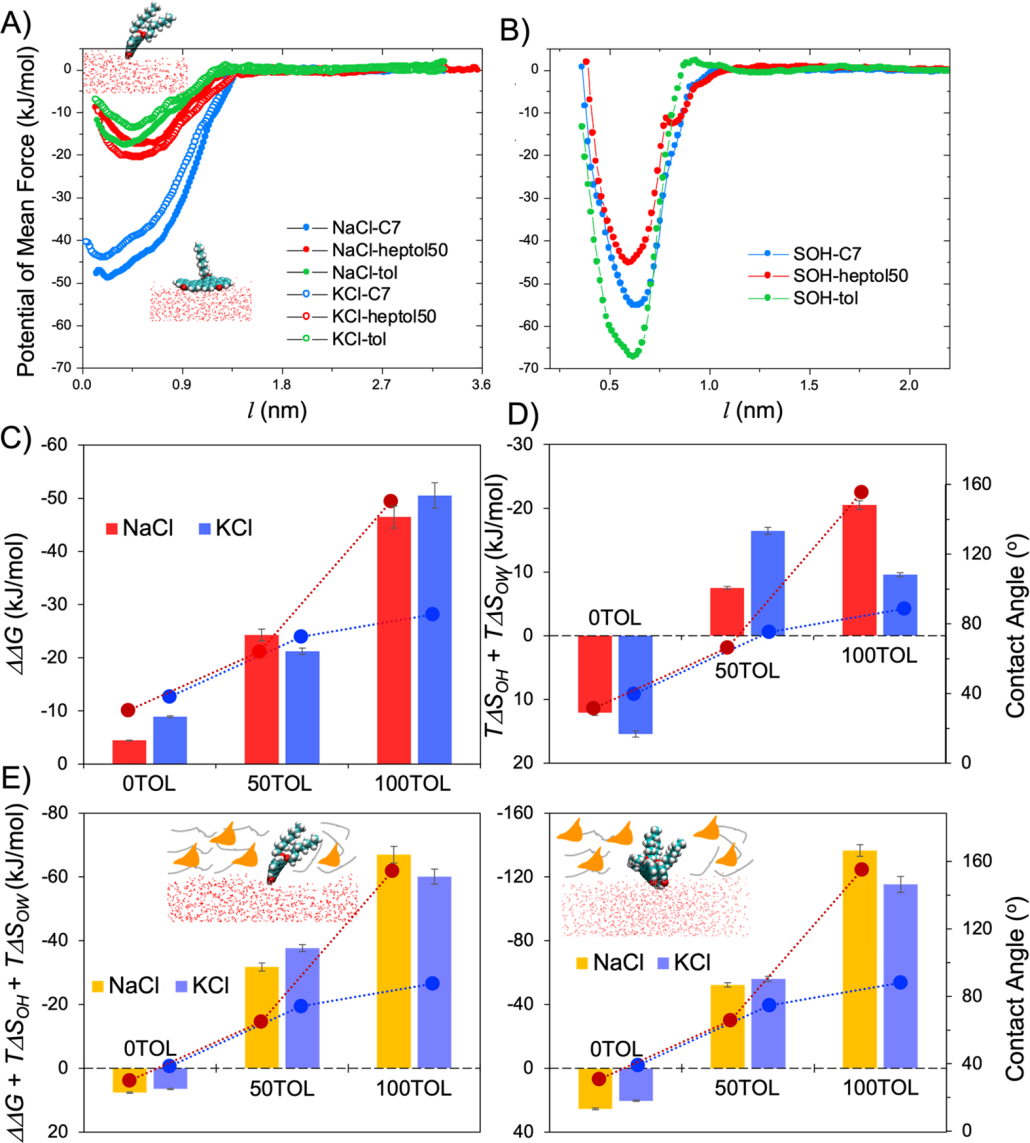
(A) Potential of mean force (PMF) profiles along the *Z* direction (perpendicular to the interface) experienced by one PAH molecule moving toward the solvent/KCl (empty circles) and solvent/NaCl (filled circles). (B) PMF profiles obtained as the PAH approaches the solvent–hydrate interfaces. The distance *l* is calculated between the center of mass of the PAH molecule and the position of the relevant interfaces. (C) Relative binding free-energy difference *ΔΔG* (bars) and (D) entropy changes associated with solvation of one PAH at the solvent–brine (*TΔS*_OW_) and solvent–hydrate (*TΔS*_OH_) interfaces with the contact angles obtained for KCl and NaCl droplets (blue and red filled circles, respectively) on the hydrate surface. (E) Sum of binding free-energy difference and entropy changes (bars) associated with solvation of one PAH monomer (left) and PAH dimer (right) at the solvent–brine and solvent–hydrate interfaces with the relevant contact angles (blue and red filled circles for KCl and NaCl droplets, respectively) on the hydrate surface. The results were obtained for various solvents. Error bars for the quantification of relative binding free-energy difference derived from PMFs are estimated from bootstrap analysis implemented in GROMACS^50^ while the ones for calculating configurational entropy changes upon oil solvation are obtained from three independent simulations. The error bars are smaller than the symbols used to illustrate the contact angle data.

In an attempt to identify molecular features that correlate with these results, well-tempered metadynamics simulations^46,47^ were conducted. This method allows us to estimate the free-energy (FE) landscape sampled as the orientational angle formed between the vector normal to the PAH polyaromatic core and the surface normal changes along the *Z* direction (details provided in the SI). In Figure S3, the FE landscapes show that the most stable structure of the PAH at the heptane–brine interface (blue) maintains the polyaromatic PAH core nearly parallel to the interface (∼17°) while the PAH preferentially orients its polyaromatic core perpendicular to the heptol50/brine (red) and toluene/brine (green) interfaces. It is known that the π–OH interaction between aromatic compounds and water is attractive (−3.16 kcal/mol)^48^ and that this interaction becomes even more attractive when K^+^/Na^+^/Ca^2+^ and Cl^–^ ions are present.^41^ These effects explain why we observe mainly parallel orientations of the polyaromatic PAH core at the pure heptane/brine interface. Increasing toluene content in the solvent seems to cause a competition between toluene and PAH for access to the interface, resulting in the preferential perpendicular orientation of the polyaromatic PAH core when heptol50 or pure toluene are used. This could explain why PAH experiences a much stronger attraction to the pure heptane–brine interface (blue) compared to the other interfaces considered here.

We also conducted umbrella sampling simulations^49^ and employed the weighted histogram analysis method^50^ to examine the interactions between PAH and solvent–hydrate interfaces (see Figure 3B). The results show that the attractive interactions between PAH and the solvent–hydrate interface increase in the following order: heptol50 (red) < pure heptane (blue) < pure toluene (green), which may seem inconsistent with the order of the attractive interactions between the PAH and the solvent–brine interface (see Figure 3A). Via visual inspection of the PAH orientation at the solvent–hydrate interface, we observe that the PAH mostly orients its polyaromatic core nearly perpendicular to the interface in all solvents considered, including pure heptane, which is contrary to the results obtained at the solvent–brine interface (see Figure S3). This is possibly due to the negligible π–OH interactions between PAH and the water molecules contained in the clathrate hydrates structures, which assemble into crystalline polyhedral cages stabilized by hydrogen bonds. Quantitative analysis of the PAH orientation at the solvent–hydrate interface reveals that the PAH preferentially orients its polyaromatic core closer to the toluene–hydrate interface (∼59°) compared to other interfaces (∼76°), suggesting stronger attractions between the PAH and the toluene–hydrate interface. The PAH–heptane/hydrate interactions are more attractive than the PAH–heptol50/hydrate ones probably because of the favorable π–π interactions between PAH and toluene in the bulk phase. It is worth noting that while the effective attraction between PAH and pure heptane/brine interface is comparable to the one between PAH and the pure heptane–hydrate interface, the attraction between PAH and solvent–hydrate interfaces is much stronger compared to the corresponding ones at the solvent–brine interfaces when toluene is present.

To gain a quantitative understanding of the interactions between PAHs and solvent–brine or solvent–hydrate interfaces, we calculated the binding free energy (*ΔG*) for one PAH molecule at the interface as the difference of free energies in the bound and unbound states (see details in the SI):^51^
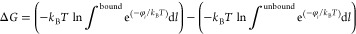
1where *φ*_*i*_ is the PMF value associated with the *i*th bin along the distance *l*. The relative binding free-energy difference (*ΔΔG*) for the PAH–solvent/hydrate and the PAH–solvent/brine interfaces is then calculated as
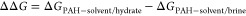
2

In Figure 3C, we report the *ΔΔG* obtained for the various systems considered. We observe a direct relationship between *ΔΔG* (columns) and contact angle (filled circles) as the toluene volume fraction increases for NaCl (red) and KCl (blue) droplets; specifically, the contact angle increases as the *ΔΔG* decreases. However, because the contact angle of the NaCl droplet is much larger than that of the KCl droplet even though the *ΔΔG* obtained for the pure toluene/NaCl system is larger, we conclude that the *ΔΔG* alone cannot explain changes in clathrate hydrate wettability for the systems considered here.

Because of the significant entropic contribution in modulating the solvation of complex molecules,^52−54^ we computed the configurational entropy, *S*_L_, of PAH in solvents, at solvent–hydrate and at solvent–brine interfaces. We implemented the method developed by Schlitter and others,^55−58^ according to whom *S*_L_ is quantified by the covariance of the Cartesian coordinates of atoms of one PAH molecule via^52^
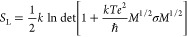
3

The entropy change because of the reorganization of PAH in the presence of solvent at density ρ can be estimated as *ΔS* = *S*_L_(ρ) – *S*_L_(ρ = 0). We report the sum of the entropy change *TΔS*_OH_ + *TΔS*_OW_ (columns in Figure 3D) associated with solvation of one PAH molecule in various solvents at the solvent–hydrate and solvent–brine interfaces at 274 K and 3.45 MPa. Note that the lower the entropy change upon solvation, the more inflexible the PAH becomes at the interfaces, suggesting possibly higher efficiency in preventing the spreading of the brine droplets on the hydrate surface, leading to higher contact angles. The results show a correlation between *TΔS*_OH_ + *TΔS*_OW_ (columns) and contact angles (filled circles) for NaCl droplets (red); i.e., the contact angle increases as *TΔS*_OH_ + *TΔS*_OW_ decreases. Notwithstanding these notes, a correlation is not observed for KCl droplets (blue).

However, on combining the relative binding free-energy difference and entropy (*ΔΔG* + *TΔS*_OH_ + *TΔS*_OW_), we observe a direct correlation for all systems considered; i.e., the lower the value of *ΔΔG* + *TΔS*_OH_ + *TΔS*_OW_ (columns), the higher the contact angle (filled circles) (see Figure 3E, left). These observations suggest that quantifying *ΔΔG* + *TΔS*_OH_ + *TΔS*_OW_ for one PAH molecule could be useful for predicting changes in clathrate hydrate wettability in the simultaneous presence of PAHs, aromatic contents in solvent, and various salts.

Because in many applications PAHs are expected to agglomerate, one could question whether the promising results presented in Figure 3E, left, remain applicable when PAH nanoaggregates are considered. In fact, Pauchard et al.^29,59,60^ proposed that asphaltene molecules consisting primarily of one PAH (“island”) or multiple PAHs (“archipelago”), as observed by atomic force microscopy and scanning tunnelling microscopy,^18^ adsorb “flat on” the solvent–water interface as a monomer, while nanoaggregates do not adsorb as readily because of the protruding aliphatic chains. These propositions, however, seem to be at odds with several other studies, which invoke the importance of strong π–π stacking interactions between PAHs.^61^ For example, Schneider et al.^14^ showed that hexa(2,2′-dipyridylamino)hexabenzocoronene (HPAHBC) orients its PAH core parallel (∼10°) to the water surface. As the number of HPAHBC molecules on the water surface increases, the aggregates form and adopt a parallel π–π stacking with the PAH core plane forming a much larger tilt angle (90°). The results obtained for the systems of PAH aggregates (see Figure S4) and the ones of PAH monomer in aromatic solvents (see Figure S3) considered in the present work seem consistent with the latter observations. Although more extensive data sets should be used to test whether the correlation proposed here has predictive capabilities, in Figure 3E, right, we report *ΔΔG* + *TΔS*_OH_ + *TΔS*_OW_ for two PAH molecules that aggregated yielding a PAH dimer. The results were obtained for NaCl (yellow) and KCl (blue) brines and various solvents. We find it promising that a correlation similar to the one obtained for individual PAH molecules exists also between *ΔΔG* + *TΔS*_OH_ + *TΔS*_OW_ for the PAH dimer and the brine droplet contact angles.

In conclusion, our simulations demonstrate that the interplay of PAHs, aromatic content, and salt-specific effects impacts significantly the interfacial properties of PAHs, and specifically their ability to influence the wettability of clathrate hydrates. In some cases, spontaneous rolling of NaCl and CaCl_2_ brine droplets was observed on PAH-covered hydrate surfaces (the interested reader is referred to Movie S1 reported in the SI). More importantly, this study reveals a direct correlation between the wetting properties and a combination of binding free-energy difference and entropy changes upon solvation for PAHs at solvent–brine and solvent–hydrate interfaces. While this correlation holds for the systems considered in this work, more extensive studies, conducted for a variety of salts and solvents, should be conducted, perhaps guided by a design of experiments approach, to assess whether this correlation is general. Our results emphasize the importance of molecular thermodynamics in explaining the molecular mechanisms governing wettability alteration, phenomena that are of importance for further developing applications of PAHs in optical, microfluidics, manufacturing technologies, as well as in hydrate management.

## Supporting Information Available

Movie S1: spontaneous rolling of brine droplets on the PAH-covered hydrate surface (MOV)
Details about simulation models, algorithms, implementation methods, calculation procedures, and results for properties of interest such as contact angle of a brine droplet on the bare C1–C2 hydrate surface, contact angle of a water droplet on the C1–C2 hydrate surface covered with PAHs when salt ions are not present, estimated free energy as a function of the orientational angle for a PAH molecule at solvent–brine interfaces, simulation snapshots showing the configurations of PAH aggregation at solvent–brine interfaces, potential of mean force as experienced by PAH nanoaggregates moving toward solvent–brine and solvent–hydrate interfaces, relative binding free-energy difference *ΔΔG*, and sum of entropy changes associated with solvation of the PAH nanoaggregates at the solvent–brine and solvent–hydrate interfaces (PDF)

## Supplementary Material

jz2c01846_si_001.mov
