# Supplementary material for: Wetting Properties of Clathrate Hydrates in the Presence of Polycyclic Aromatic Compounds: Evidence of Ion-Specific Effects
Source: J Phys Chem Lett. 2022 Aug 25;13(34):8200–6. doi: 10.1021/acs.jpclett.2c01846 (PMC9442800; doi:10.1021/acs.jpclett.2c01846)
Supplement: Supplementary file 2 — jz2c01846_si_002.pdf [file jz2c01846_si_002.pdf]

Wetting Properties of Clathrate Hydrates in the Presence of Polycyclic Aromatic Compounds: Evidence of Ion-Specific Effects

Anh Phan[1*], Michail Stamatakis[2], Carolyn A. Koh[3], and Alberto Striolo[2,4*]

[1]Department of Chemical and Process Engineering, Faculty of Engineering and Physical Sciences, University of Surrey, Guildford, Surrey GU2 7XH, UK

[2]Department of Chemical Engineering, University College London, London, WC1E 7JE, UK

[3]Center for Hydrate Research, Chemical & Biological Engineering Department, Colorado School of Mines, Golden, Colorado 80401, United States

[4]School of Chemical, Biological and Materials Engineering, University of Oklahoma, Norman, Oklahoma 73019, United States

Supplementary Information

*Corresponding Authors

a.phan@surrey.ac.uk

astriolo@ou.edu

Methods and Algorithms

Model Setup. Our simulation setup mimics the sessile contact angle measurement for a water droplet on a hydrate surface coated with polycyclic aromatic hydrocarbons (PAHs) in the presence of a liquid hydrocarbon. In this study, Violanthrone-79, which has a single polyaromatic core, aliphatic chains, and functional groups with heteroatoms such as oxygen, was chosen as representative model PAH (see **Figure 1A**). The sII hydrate structure was employed as the solid substrate to describe the methane–ethane (C1–C2) hydrates considered here. To construct the hydrate configurations, we adopted the sII unit cell from Takeuchi et al.[1] Tiling the unit cell in *X* and *Y* directions, the *X*, *Y* and *Z* dimensions of the hydrate substrate were 10.386, 5.193, and 1.731 nm, respectively. To build the C1–C2 hydrate surface coated with PAHs, we placed 28 PAH molecules on top of the hydrate surface, and then we equilibrated the system at 274 K and 3.45 MPa until it achieved equilibrium. We present the 2D density profiles obtained for PAHs adsorbed on the hydrate surface (see **Figure 1B**) to show the distribution of PAH aggregation.

The simulation box length was ~11.5 nm in the *Z* direction, with the hydrate substrate aligned parallel to the *X*–*Y* plane. The hydrate substrate model becomes infinitely long along the *X* and *Y* directions when applying periodic boundary conditions in all directions. Upon equilibration, we used a cylindrical water droplet periodic along the *Y* direction – the cylinder axis, as this configuration guarantees that the line tension of the three-phase boundary does not influence contact angle estimates from simulations.[2-4]

The ~3.5 wt % KCl, NaCl, and $CaCl_2$ cylindrical droplets (3.5 wt % is the bulk concentration of seawater), periodic along the Y direction, were then situated on top of the PAH-covered hydrate surface, before the simulation box was filled with the solvent, which consists of n-heptane and toluene at various relative concentrations, i.e., pure heptane, heptol75 (75 vol % heptane – 25 vol % toluene), heptol50 (50 vol % heptane – 50 vol % toluene), and pure toluene (see **Figure 1C**). The volume fraction was calculated based on the number of heptane/toluene molecules (as shown in **Table S1**) and their densities at simulation conditions. In **Table S1**, we report the compositions of the simulated systems.

Force Fields. Water was represented by the TIP4P/Ice model,[5] which has been used successfully in studying hydrate systems.[6-8] Methane, ethane, and n-heptane were represented by employing the united-atom version of the TraPPE-UA force field,[9] which correctly describes the critical properties and the vapor-liquid coexistence of linear alkanes far from the critical point. PAH and toluene were modelled implementing the General Amber Force Field (GAFF), often employed to study cyclic, organic, and pharmaceutical compounds containing H, C, N, O, S, P, and halogens.[10-11] Na^+, K^+ and Cl^- ions were modeled as charged Lennard-Jones (LJ) spheres by using the parameters suggested by Dang.[12] Ca^{2+} ions were modeled as charged LJ spheres by employing the parameters reported by Netz et al.[13]

All non-bonded interactions were described using 12−6 Lennard-Jones (LJ) potentials with the cutoff distance of 1.4 nm. The electrostatic interactions were modeled by the Coulombic potential with long-range corrections treated using the particle–particle particle–mesh (PPPM) approach[14] with a grid spacing in the direct lattice of 0.12 nm and a fourth-order B-spline interpolation for the gridded charge array.[9, 15] We utilized the Lorentz−Berthelot combining rules to quantify unlike LJ interactions.[16] Previous computational studies suggest that distance cutoff of 1.4 nm used for all non-bonded interactions and the PPPM approach employed for the treatment of long-range corrections together with the combination of GAFF force field for modelling cyclic and organic molecules, the TIP4P/Ice model for water and TraPPE-UA force field for simulating linear alkanes are appropriate, achieving excellent agreement against experimental studies.[2, 6, 17]

Implementation. Equilibrium MD simulations were performed employing the GROMACS package,[18] version 2016.3. To quantify contact angles on PAH-adsorbed hydrate surfaces, we first performed simulations in the NVT canonical ensemble (constant volume, temperature, and number of particles) for 1 ns to relax the initial configurations, while the hydrate layer was kept rigid. We then carried out simulations within the NPT ensemble (T = 274 K and P = 3.45 MPa) implementing the Nose-Hoover thermostat and the Berendsen/Parrinello-Rahman barostat.[18] We applied the pressure coupling algorithm only along the Z direction, which allows X and Y dimensions of the simulation box to remain fixed.

Using the leapfrog algorithm, the equations of motion were solved with the time step of 1.0 fs.[18] We implemented a harmonic restraint force constant of 2000 kJ/mol/nm on water as well as C1–C2 molecules

in the hydrate phase to tether them to their initial positions[18] while other molecules in the system were allowed to move freely. In our recent study, in which these constraints were applied on the hydrates (water/guests), we showed good agreement against experimental observations, suggesting that this approach yields reliable outcomes.[2] We conducted each NPT simulation for 500–700 ns until both fluids appeared to be stable, and the droplet shape remained unchanged within a simulation time interval of 20 ns.

Contact Angle Calculation

We extracted contact angles from 2D density profiles obtained for the simulated brine droplets (see the insets in **Figure 2**). The iso-density contours at density ρ_o, obtained as halfway between the water density in the hydrocarbon phase and the water bulk density,[3] were used to determine the contact angle for all systems considered. Once the droplet contours were identified following the procedure described in previous studies,[2-4] we fit them with a circular function. The droplet base was identified at the second hydration layer away from the hydrate surface, and the slopes of the tangent lines on both sides of the droplet were determined to calculate the contact angle.

Table S1. Composition of the systems simulated in this work.

<table>
<tr><th></th><th>N_{K+}</th><th>N_{Na+}</th><th>N_{Ca2+}</th><th>N_{Cl-}</th><th>$N_{H2O(droplet)}$</th><th>$N_{n\text{-}heptane}$</th><th>$N_{toluene}$</th></tr>
<tr><td>KCl/pure heptane</td><td rowspan="4">12</td><td rowspan="4"></td><td rowspan="4"></td><td rowspan="4">12</td><td rowspan="4">1070</td><td>1900</td><td>0</td></tr>
<tr><td>KCl/heptol75</td><td>1424</td><td>650</td></tr>
<tr><td>KCl/heptol50</td><td>950</td><td>1300</td></tr>
<tr><td>KCl/pure toluene</td><td>0</td><td>2600</td></tr>
<tr><td>NaCl/pure heptane</td><td rowspan="4"></td><td rowspan="4">12</td><td rowspan="4"></td><td rowspan="4">12</td><td rowspan="4">1070</td><td>1900</td><td>0</td></tr>
<tr><td>NaCl/heptol75</td><td>1424</td><td>650</td></tr>
<tr><td>NaCl/heptol50</td><td>950</td><td>1300</td></tr>
<tr><td>NaCl/pure toluene</td><td>0</td><td>2600</td></tr>
<tr><td>$CaCl_2$/pure heptane</td><td rowspan="4"></td><td rowspan="4"></td><td rowspan="4">6</td><td rowspan="4">12</td><td rowspan="4">1070</td><td>1900</td><td>0</td></tr>
<tr><td>$CaCl_2$/heptol75</td><td>1424</td><td>650</td></tr>
<tr><td>$CaCl_2$/heptol50</td><td>950</td><td>1300</td></tr>
<tr><td>$CaCl_2$/pure toluene</td><td>0</td><td>2600</td></tr>
</table>

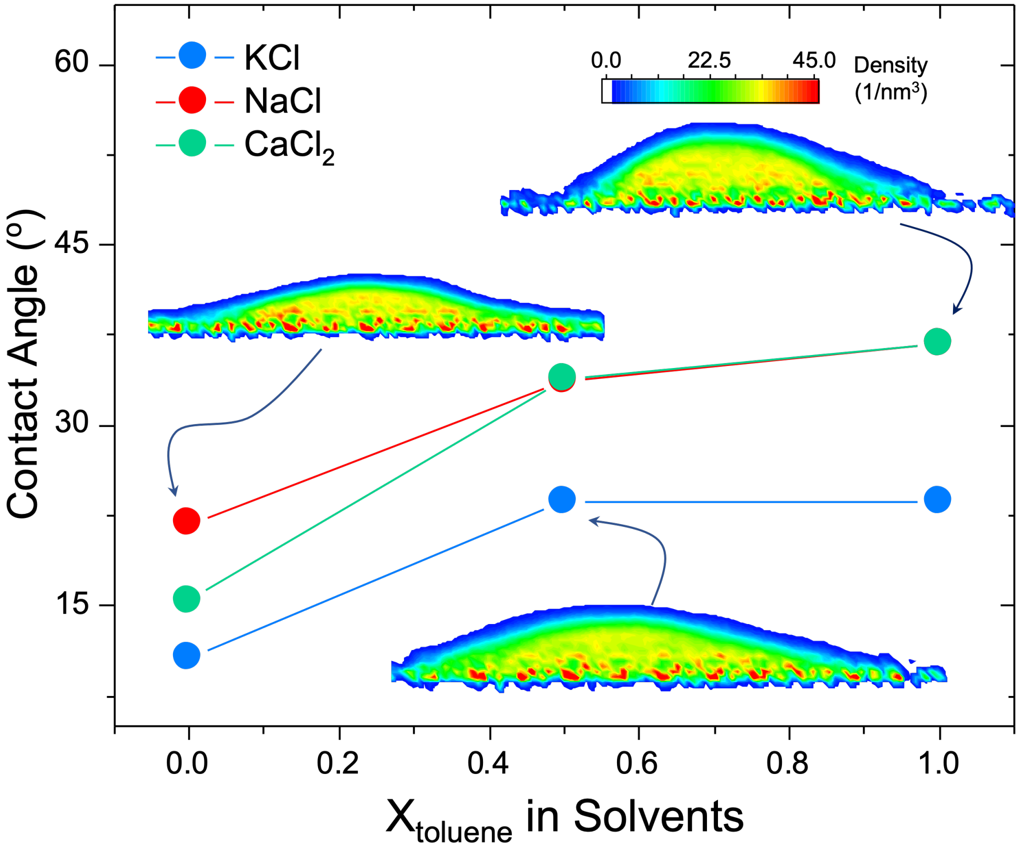

Figure S1. Contact angle estimation of one brine droplet deposited on the C1–C2 hydrate surface in the absence of PAHs. The results were obtained for the aqueous droplets containing ~3.5 wt % KCl (blue), NaCl (red), and $CaCl_2$ (green) in solvents of varying toluene volume fraction. The insets show 2D density profiles of various brine droplets on the hydrate surfaces. The colour bar shows water density in the units of 1/nm^3. Error bars, which were obtained from three independent simulation runs for contact angle estimation, are smaller than the symbols used to display the data.

In **Figure S1**, we present the contact angle of a brine droplet on the bare C1–C2 hydrate surface. The results were obtained for the aqueous droplets containing ~3.5 wt % KCl (blue), NaCl (red), and $CaCl_2$ (green) in solvents of varying toluene volume fraction. The results, in general, suggest that the contact angle of the brine droplet increases when the toluene content is increased. An insignificant difference is observed in the contact angle values as a function of salt type (the results remain in the range of 10–36°), especially when compared to similar results obtained for brine droplets deposited on the PAH-covered hydrate surface (see **Figure 2**).

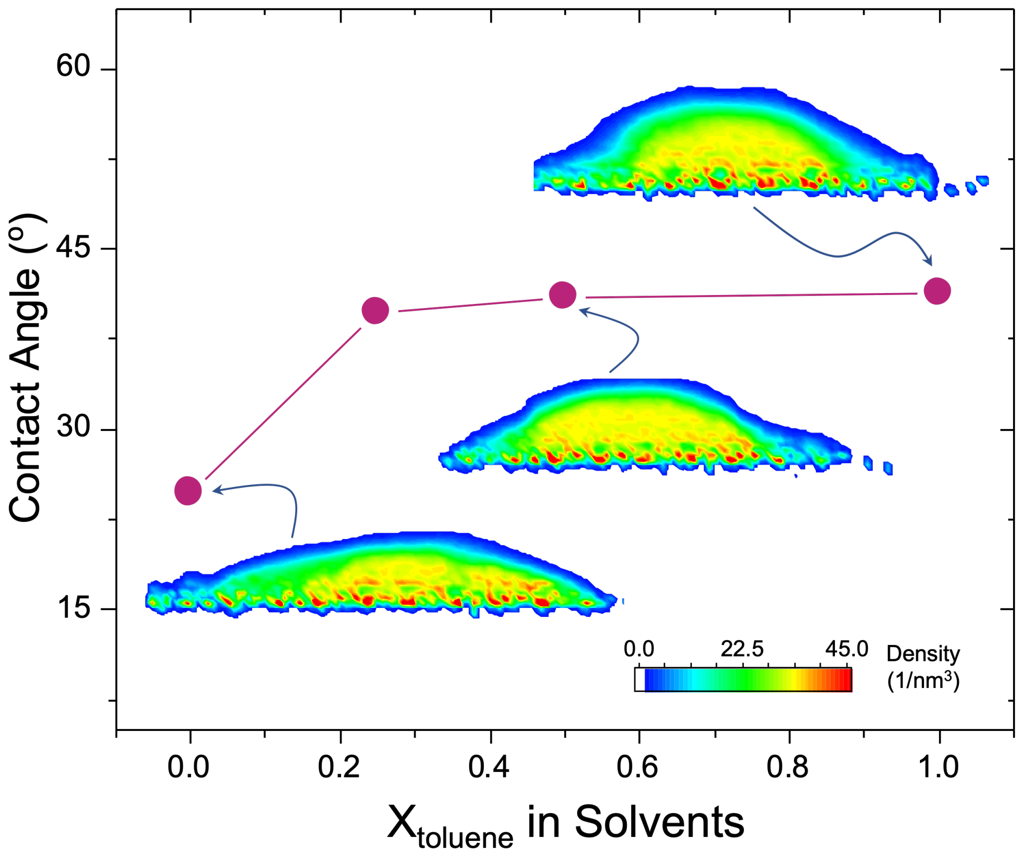

Figure S2. Contact angle estimation of one water droplet deposited on the C1–C2 hydrate surface covered with PAHs and immersed in oil solvents. The insets show 2D density profiles of various water droplets on the hydrate surfaces in solvents of varying toluene volume fraction. The colour bar shows water density in the units of $1/nm^3$. Error bars, which were obtained from three independent simulation runs for contact angle estimation, are smaller than the symbols used to display the data.

In **Figure S2**, we show the contact angle of a water droplet on the C1–C2 hydrate surface covered with PAHs when salt ions are not present. The results were obtained for the aqueous droplets immersed in solvents of varying toluene volume fraction. The results, in general, suggest that the contact angle of the water droplet increases when the toluene content is increased. An insignificant difference is observed in the contact angle values as a function of toluene volume fraction (the results remain in the range of 24–41°), especially when compared to similar results obtained for brine droplets deposited on the PAH-covered hydrate surface (see **Figure 2**).

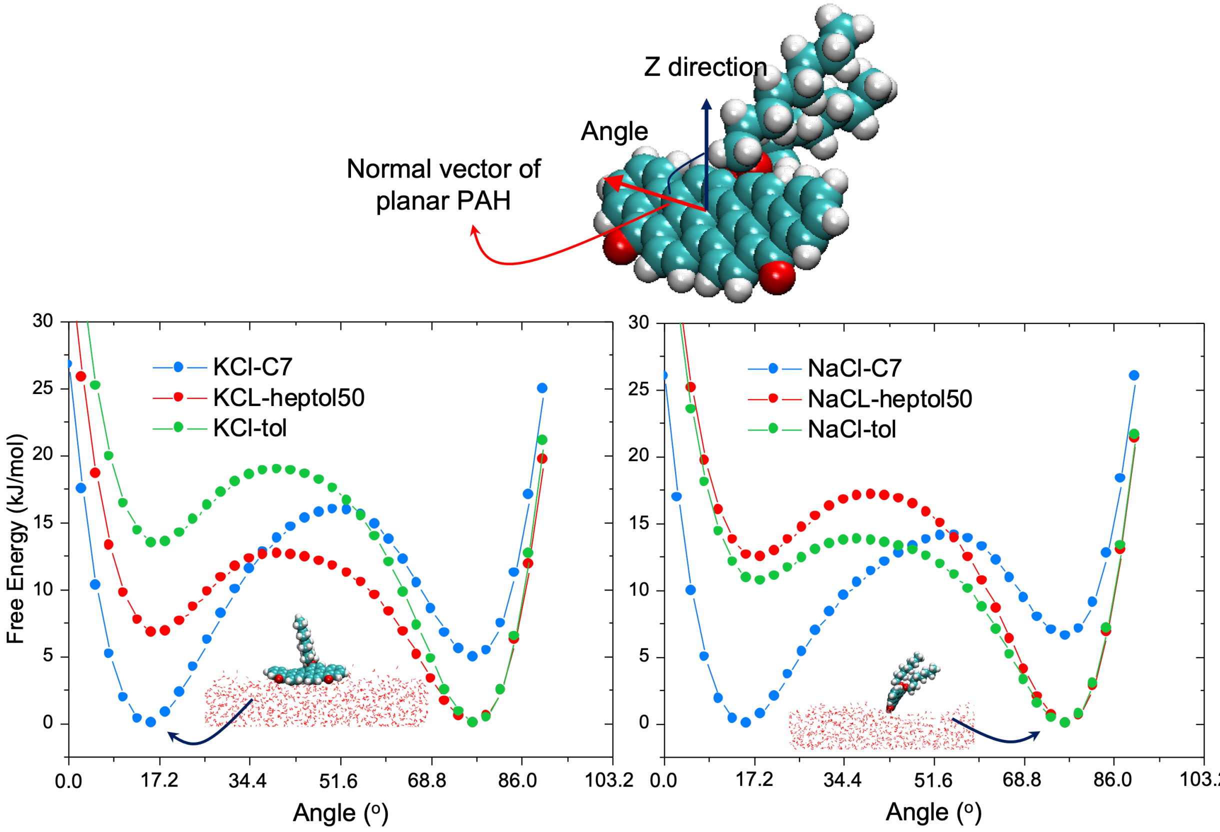

Figure S3. Free energy profile as a function of the orientational angle for one PAH molecule at solvent/KCl (left) and solvent/NaCl (right) interfaces. The top panel shows a diagram describing the orientational angle formed by the normal vector of the polyaromatic core of the PAH and the direction perpendicular to the interface (*Z* direction). The results were obtained for various solvents such as pure heptane (blue), heptol50 (red), and pure toluene (green).

Employing well-tempered metadynamics,[19-20] we estimated free energy (FE) profiles as a function of the orientational angle for PAH molecules at solvent/brine interfaces in **Figure S3**. The FE landscapes show that the most stable orientation for the PAH at the heptane/brine interface (blue) corresponds to the PAH polyaromatic core parallel to the interface while the PAH preferentially orients its polyaromatic core perpendicular to the heptol50/brine (red) and toluene/brine (green) interfaces.

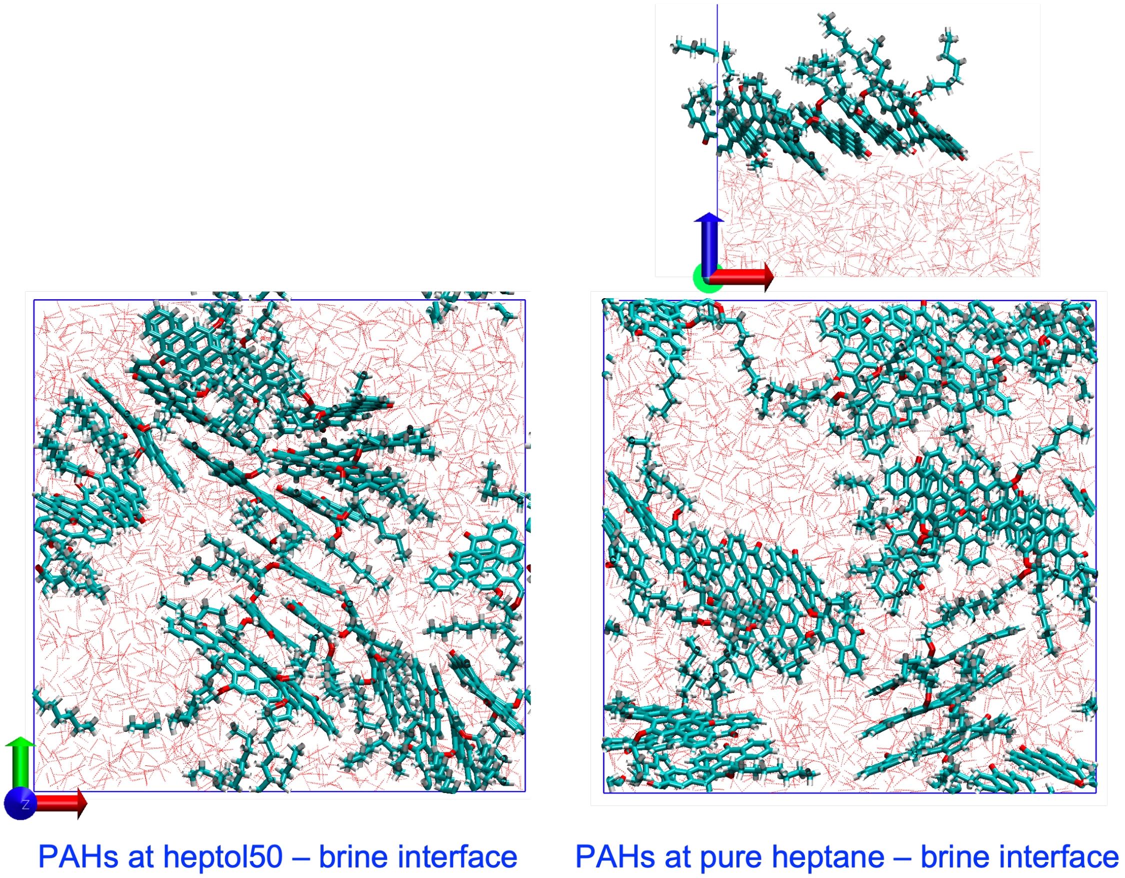

Figure S4. Simulation snapshots demonstrate the configuration of 28 PAH molecules at heptol50/NaCl (left) and pure heptane/NaCl (right) interfaces, showing the most frequent orientation and packing adopted by PAH molecules. Red wireframes symbolize water phase while sodium and chlorine molecules in water are not shown for clarity. Red, white, and cyan sticks represent oxygen, hydrogen, and carbon atoms of PAH molecules, respectively.

In **Figure S4**, we show simulation snapshots to illustrate the configuration of 28 PAH molecules at heptol50/NaCl (left) and pure heptane/NaCl (right) interfaces, highlighting the most frequent orientation and packing structures adopted by the PAH molecules. With an increasing number of PAH molecules, the results show that the molecules aggregate via parallel π–π stacking with the PAH core plane forming a much larger tilt angle (90°) even at the pure heptane/brine interface, on which one single PAH monomer orients its PAH core parallel to the water surface (see **Figure S3**). This observation agrees with results reported by Schneider et al.[21] for the configuration of core-rim PAH monomer hexa(2,2'-dipyridylamino)hexabenzocoronene (HPAHBC) molecules on the water surface.

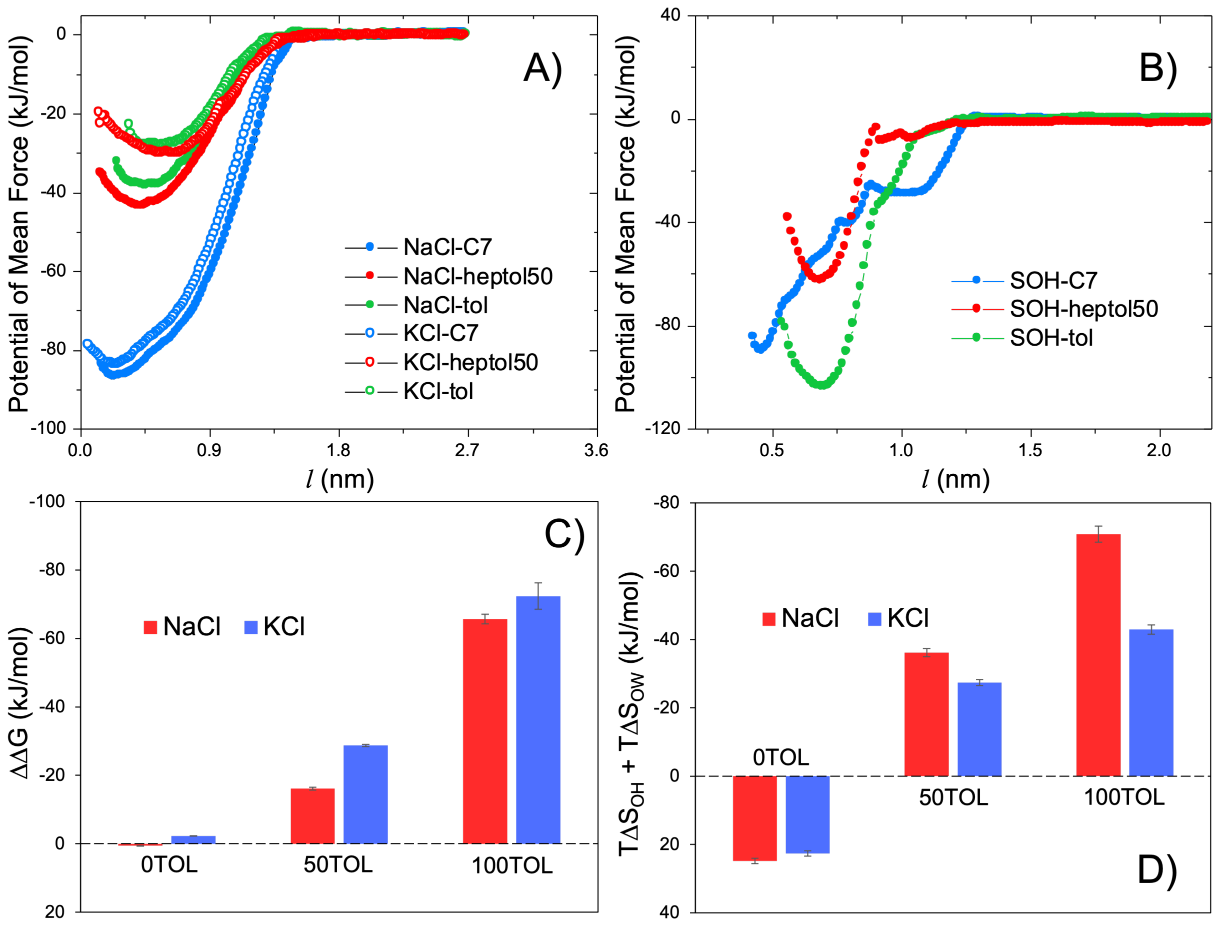

Figure S5. A) Potential of mean force profiles along the *Z* direction (perpendicular to the interface) as experienced by PAH nanoaggregates (PAH dimer) moving toward the solvent/KCl (empty circles) and solvent/NaCl (filled circles), and B) solvent/hydrate interfaces. The distance *l* between the centre of mass of the PAH nanoaggregates and the interface. C) Relative binding free energy difference *ΔΔG* for the PAH nanoaggregates at the solvent/brine and solvent/hydrate interfaces. D) Sum of entropy changes associated with solvation of the PAH nanoaggregates at the solvent/brine and solvent/hydrate interfaces. The results were obtained for pure heptane, heptol50 and pure toluene.

In **Figure S5**, panel A, we present the potential of mean force (PMF) profiles experienced by PAH-PAH dimers as they approach the solvent/brine interface along the *Z* direction, perpendicular to the interface. The results are shown for pure heptane (blue) and pure toluene (red). We considered only those systems with KCl (empty circles) and NaCl (filled circles), since the wetting behaviours of NaCl and $CaCl_2$ droplets on the hydrate surfaces are similar for all solvents considered (see **Figure 2**). The PMF profiles were obtained as functions of the distance *l* between the centre of mass of the PAH molecule and the interfaces. The results, in general, show an effective attraction between PAH molecules and interfaces. We observe

negligible salt-specific effects on the PMF between PAH-PAH dimers and heptane/brine interface. On the other hand, the salts show moderate impact on the PMFs between PAH-PAH dimers and solvent/brine interfaces when toluene is present. The PMFs also strongly depend on the solvent composition; specifically, the PAH experiences much stronger attraction to the pure heptane/brine interface compared to pure toluene/brine interfaces, at distances at which the PAH starts to interact with the interface.

In **Figure S5**, panel B, we report the PMF profiles experienced by PAH nanoaggregates as they reach the solvent/hydrate interface. The simulation results show that the attractive interactions increase in the following order: pure toluene (red) < pure heptane (blue).

We show results for $\Delta\Delta G$ and ($T\Delta S_{OH}$ + $T\Delta S_{OW}$) obtained for PAH nanoaggregates in various systems in **Figure S5**, panel C and D, respectively. We do not observe a direct correlation between the relative binding free energy difference $\Delta\Delta G$ and the wetting behaviour of brine droplets on the PAH-adsorbed hydrate surfaces immersed in oil solvents, as discussed in the main text.

References

(1) Takeuchi, F.; Hiratsuka, M.; Ohmura, R.; Alavi, S.; Sum, A. K.; Yasuoka, K., Water Proton Configurations in Structures I, Ii, and H Clathrate Hydrate Unit Cells. *J Chem Phys* **2013**, *138*, 124504

(2) Phan, A.; Stoner, H. M.; Stamatakis, M.; Koh, C. A.; Striolo, A., Surface Morphology Effects on Clathrate Hydrate Wettability. *J Colloid Interf Sci* **2022**, *611*, 421-431.

(3) Le, T. T. B.; Striolo, A.; Cole, D. R., Supercritical Co2 Effects on Calcite Wettability: A Molecular Perspective. *J Phys Chem C* **2020**, *124*, 18532-18543.

(4) Le, T. T. B.; Divine-Ayela, C.; Striolo, A.; Cole, D. R., Effects of Surface Contamination on the Interfacial Properties of Co2/Water/Calcite Systems. *Phys Chem Chem Phys* **2021**, *23*, 18885-18892.

(5) Abascal, J. L. F.; Sanz, E.; Fernandez, R. G.; Vega, C., A Potential Model for the Study of Ices and Amorphous Water: Tip4p/Ice. *J Chem Phys* **2005**, *122*, 234511.

(6) Phan, A.; Stamatakis, M.; Koh, C. A.; Striolo, A., Correlating Antiagglomerant Performance with Gas Hydrate Cohesion. *ACS Appl Mater Interfaces* **2021**, *13*, 40002-40012.

(7) Walsh, M. R.; Koh, C. A.; Sloan, E. D.; Sum, A. K.; Wu, D. T., Microsecond Simulations of Spontaneous Methane Hydrate Nucleation and Growth. *Science* **2009**, *326*, 1095-1098.

(8) Phan, A.; Schlösser, H.; Striolo, A., Molecular Mechanisms by Which Tetrahydrofuran Affects Co2 Hydrate Growth: Implications for Carbon Storage. *Chem Eng J* **2021**, *418*, 129423.

(9) Martin, M. G.; Siepmann, J. I., Transferable Potentials for Phase Equilibria. 1. United-Atom Description of N-Alkanes. *J Phys Chem B* **1998**, *102*, 2569-2577.

(10) Wang, J. M.; Wolf, R. M.; Caldwell, J. W.; Kollman, P. A.; Case, D. A., Development and Testing of a General Amber Force Field. *J Comput Chem* **2004**, *25*, 1157-1174.

(11) Case, D. A., et al., Amber 14. University of California, San Francisco, 2014.

(12) Dang, L. X., Mechanism and Thermodynamics of Ion Selectivity in Aqueous-Solutions of 18-Crown-6 Ether - a Molecular-Dynamics Study. *J Am Chem Soc* **1995**, *117*, 6954-6960.
(13) Mamatkulov, S.; Fyta, M.; Netz, R. R., Force Fields for Divalent Cations Based on Single-Ion and Ion-Pair Properties. *J Chem Phys* **2013**, *138*.
(14) Eastwood, J. W.; Hockney, R. W.; Lawrence, D. N., P3m3dp - the 3-Dimensional Periodic Particle-Particle-Particle-Mesh Program. *Comput Phys Commun* **1980**, *19*, 215-261.
(15) Vassetti, D.; Pagliai, M.; Procacci, P., Assessment of Gaff2 and Opls-Aa General Force Fields in Combination with the Water Models Tip3p, Spce, and Opc3 for the Solvation Free Energy of Druglike Organic Molecules. *J Chem Theory Comput* **2019**, *15*, 1983-1995.
(16) Allen, M. P.; Tildesley, D. J., Computer Simulation of Liquids. Oxford University Press: Oxford, UK, 2004.
(17) Bui, T.; Phan, A.; Monteiro, D.; Lan, Q.; Ceglio, M.; Acosta, E.; Krishnamurthy, P.; Striolo, A., Evidence of Structure-Performance Relation for Surfactants Used as Antiagglomerants for Hydrate Management. *Langmuir* **2017**, *33*, 2263-2274.
(18) Abraham, M. J.; Murtola, T.; Schulz, R.; Páll, S.; Smith, J. C.; Hess, B.; Lindahl, E., Gromacs: High Performance Molecular Simulations through Multi-Level Parallelism from Laptops to Supercomputers. *SoftwareX* **2015**, *1*, 19-25.
(19) Barducci, A.; Bussi, G.; Parrinello, M., Well-Tempered Metadynamics: A Smoothly Converging and Tunable Free-Energy Method. *Phys Rev Lett* **2008**, *100*.
(20) Bonomi, M., et al., Plumed: A Portable Plugin for Free-Energy Calculations with Molecular Dynamics. *Comput Phys Commun* **2009**, *180*, 1961-1972.
(21) Liu, X.; He, M.; Calvani, D.; Qi, H.; Gupta, K.; de Groot, H. J. M.; Sevink, G. J. A.; Buda, F.; Kaiser, U.; Schneider, G. F., Power Generation by Reverse Electrodialysis in a Single-Layer Nanoporous Membrane Made from Core-Rim Polycyclic Aromatic Hydrocarbons. *Nat Nanotechnol* **2020**, *15*, 307-312.
